# Kinematics of Maximal Speed Sprinting With Different Running Speed, Leg Length, and Step Characteristics

**DOI:** 10.3389/fspor.2019.00037

**Published:** 2019-09-26

**Authors:** Kenji Miyashiro, Ryu Nagahara, Kohei Yamamoto, Takahiko Nishijima

**Affiliations:** ^1^Law Course, Department of Law, Nihon Bunka University, Tokyo, Japan; ^2^National Institute of Fitness and Sports in Kanoya, Kanoya, Japan; ^3^Faculty of Health and Sports Sciences, University of Tsukuba, Ibaraki, Japan; ^4^Department of Sports and Health Sciences, Fukui University of Technology, Fukui, Japan

**Keywords:** running motion, multiple regression analysis, athletics, step frequency, biomechanics

## Abstract

This study aimed to provide multiple regression equations taking into account differences in running speed, leg length, and step characteristics to predict kinematics of maximal speed sprinting. Seventy-nine male sprinters performed a maximal effort 60-m sprint, during which they were videoed through the section from the 40- to 50-m mark. From the video images, leg kinematic variables were obtained and used as dependent variables for multiple linear regression equation with predictors of running speed, leg length, step frequency, and swing/support ratio. Multiple regression equations to predict leg kinematics of maximal speed sprinting were successfully obtained. For swing leg kinematics, a significant regression model was obtained to predict thigh angle at the contralateral foot strike, maximal knee flexion and thigh lift angular velocities, and maximal leg backward swing velocity (adjusted *R*^2^ = 0.194–0.378, medium to large effect). For support leg kinematics, a significant regression model was obtained to predict knee flexion and extension angular displacements, maximal knee extension velocity, maximal leg backward swing angular velocity, and the other 13 kinematic variables (adjusted *R*^2^ = 0.134–0.757, medium to large effect). Based on the results, at a given leg length, faster maximal speed sprinting will be accompanied with greater thigh angle at the contralateral foot strike, greater maximal leg backward swing velocity during the swing phase, and smaller knee extension range during the support phase. Longer-legged sprinters will accomplish the same running speed with a greater thigh angle at contralateral foot strike, greater knee flexion range, and smaller maximal leg backward swing velocity during the support phase. At a given running speed and leg length, higher step frequencies will be achieved with a greater thigh angle at contralateral foot strike and smaller knee flexion and extension ranges during the support phase. At a given running speed, leg length and step frequency, a greater swing/support ratio will be accompanied with a greater thigh angle at contralateral foot strike and smaller knee extension angular displacement and velocity during the support phase. The regression equations obtained in this study will be useful for sprinters when trying to improve their maximal speed sprinting motion.

## Introduction

Maximal speed during a 100-m race is strongly related to total race time (Slawinski et al., 2017). Therefore, maximal speed sprinting is of great importance for a 100-m race. In addition, the potential to run at greater maximal speed will improve performance for 200- and 400-m races and also the long- and triple jumps (Hanon and Gajer, 2009; Koyama et al., 2011; Panoutsakopoulos et al., 2016). Accordingly, examining the determinants of maximal speed sprinting performance is valuable not only for improving 100-m race performance but also for enhancing performance in other events.

Associations of leg kinematics and maximal speed sprinting performance have broadly been investigated (Kunz and Kaufmann, 1981; Alexander, 1989; Ae et al., 1992; Bushnell and Hunter, 2007; Ito et al., 2008; Yada et al., 2011; Toyoshima and Sakurai, 2016; Haugen et al., 2018). For joint kinematics, greater maximal running speed was associated with more extended knee joint angle at the mid-support (Yada et al., 2011), smaller knee joint angle at toe-off (Bushnell and Hunter, 2007; Yada et al., 2011), greater minimal knee joint angle during the swing phase (Ito et al., 2008), greater hip extension velocity during the support phase (Ae et al., 1992; Ito et al., 2008), and smaller knee extension velocity during the support phase (Ito et al., 2008). For segmental kinematics, greater maximal running speed was associated with greater forward lean of the shank at toe-off (Yada et al., 2011), less forward lean of the thigh at toe-off (Yada et al., 2011), higher forward lean shank angular velocity at foot strike (Toyoshima and Sakurai, 2016), and greater maximal forward lean thigh angular velocity during the support phase (Alexander, 1989). Moreover, greater maximal running speed was accompanied with greater whole leg backward swing velocity at foot strike (Ae et al., 1992) and a smaller horizontal distance between the knees at foot strike (Bushnell and Hunter, 2007; Yada et al., 2011).

Although the aforementioned previous studies provided valuable knowledge of the important kinematic features for faster maximal speed sprinting, corresponding features would likely be different based on a specificity of individuals. Theoretically, longer leg length will produce greater endpoint velocity for a given angular velocity, but longer leg length is also typically accompanied by a greater moment of inertia. Thus, differences in leg length may produce differences in kinematics for faster maximal speed sprinting. In addition to leg length, combinations of step length and frequency, which is partly affected by the leg length, are factors that influence kinematics of faster maximal speed sprinting (Toyoshima and Sakurai, 2016). Accordingly, it is essential to investigate the association of kinematics of sprinting with maximal running speed, taking into account the step characteristics in addition to the leg length. Because stride frequency is an inverse of stride time and one stride consists of the support and swing phases, there can be various combinations of support and swing times (swing/support ratio) even if the stride frequencies of two sprinters are equal to each other. Consequently, considering not only the leg length but also these step characteristics (step frequency and swing/support ratio) will improve the understanding of the kinematics of faster maximal speed sprinting.

To investigate influences of the leg length and spatiotemporal variables, in addition to running speed, on leg kinematic variables, multiple regression analyses would be useful and allow us to evaluate magnitudes of changes in kinematic variables with manipulating running speed, leg length, and spatiotemporal variables. Knowledge of difference in magnitudes of changes in kinematic variables associated with changes in running speed, leg length, and spatiotemporal variables would be of great value to coaches when training a sprinter to improve maximal speed sprinting performance. Moreover, because each of previous studies investigated relationships between maximal speed sprinting performance and kinematic variables for small number of variables (Kunz and Kaufmann, 1981; Alexander, 1989; Ae et al., 1992; Bushnell and Hunter, 2007; Ito et al., 2008; Yada et al., 2011; Toyoshima and Sakurai, 2016; Haugen et al., 2018), the data as normative information which can be used by coaches and sprinters are limited. Adopting a large number of kinematic variables therefore would provide normative information for considering faster maximal sprinting performance based on individual-specific factors.

The purpose of this study was to provide multiple regression equations taking into account differences in running speed, leg length, and step characteristics to predict kinematics of maximal speed sprinting for understanding kinematics of faster maximal speed sprinting with the differences in leg length and step characteristics. In an applied environment, sprinters and coaches are trying to improve maximal speed sprinting performance based on individual-specific factors. Therefore, the findings of this study would help to provide information which could be used to inform individual-specific features of faster maximal speed sprinting.

## Materials and Methods

### Participants

The participants were 79 male sprinters (mean ± SD: age, 20.7 ± 1.9 y; stature, 1.75 ± 0.05 m; body mass, 66.6 ± 5.0 kg; personal best 100-m time, 11.08 ± 0.42 s, ranging from 10.30 to 12.14 s). Written-informed consent was obtained from participants before participating in the study which was approved by the research ethics committee of the institute.

### Experiments

After a self-selected warm-up, the participants performed a maximal effort 60-m sprint from a two-point standing position in spiked shoes. The participants were instructed to achieve their maximal speed during the section from the 40- to 50-m mark. The participants were videoed through the section from the 40- to 50-m mark using one panning camera (EX-F1, Casio, Tokyo, Japan, 300 Hz, 512 × 384 pixels). The camera was located 1 m above the ground and perpendicular to the 45-m mark from the start and was 45 m away from the center of the running lane. The camera field of view was approximately 4 m in the horizontal direction. Reference markers were placed every meter on both sides of the running lane from the 40- to 50-m mark. To ensure appropriate digital visualization of the segment coordinates, adhesive, black or white markers were attached to anatomical landmarks on the right fifth metatarsal head, ankle, knee, and greater trochanter.

### Data Processing

Seven segment endpoints (toe, the fifth metatarsal head, heel, ankle, knee, and greater trochanter for the right leg and suprasternal) of each participant from five frames before the left leg foot strike to five frames after the next left leg foot strike (i.e., one stride, two steps) were manually digitized at 150 Hz using a Frame-DIAS system (Dkh, Tokyo, Japan). Foot strike and toe-off were visually identified three times by one examiner (all identifications being consistent). From the coordinates of the digitized endpoints and the closest four reference markers (forward and backward on both sides) in the same frame, 2-D coordinates of the endpoints in the sagittal plane were obtained. The reconstruction of the data using four reference markers was performed in reference to a previous study (Nagahara et al., 2014b). The estimated errors shown in a previous study, which was performed with similar experimental setting and used the same camera, was <9 mm (Nagahara et al., 2014b). The coordinates of the segment endpoints were smoothed using a Butterworth low-pass digital filter. The cut-off frequency (4.5–10.5 Hz) was decided using residual method proposed by Wells and Winter (1980). Using the reconstructed endpoint coordinates of the fifth metatarsal head, ankle, knee, and greater trochanter for the right leg and suprasternal, a 4-segment linked model comprising the right foot, right shank, right thigh and trunk was developed. In addition, the raw left toe coordinates at the left foot strikes before and after the investigated right leg support phase were obtained for calculating stride length.

**Figure 1:**
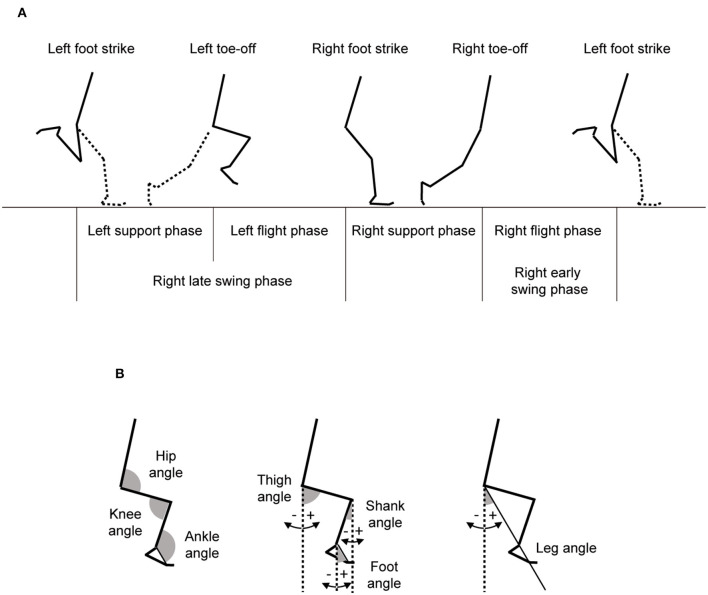
Definition of the events and phases during one stride of maximal speed sprinting and definition of joint, segment, and leg angles.

Step length was defined as half of the length between the left toe locations of consecutive two steps. Stride time was the duration from one left foot strike to the next left foot strike, with step frequency determined as the inverse of one half of stride time. Running speed was computed as the product of step length and frequency. From the left foot strike, one stride cycle was divided into four phases (left leg support phase, left leg flight phase, right leg support phase, and right leg flight phase), and the time taken for each phase was obtained (Figure 1). Moreover, the right leg swing time was computed as sum of the times for left leg support, left leg flight, and right leg flight phases. In addition, swing/support ratio was obtained dividing the right leg swing time by right leg support time, and flight/support ratio was computed by dividing the sum of the right and left leg flight times by the sum of right and left leg support times. Right leg joint and segment angles were calculated using the aforementioned 4-segment linked model as shown in Figure 1. An extension of the joints was given a positive convention. Moreover, right leg joint and segment angular velocities were computed by differentiating the corresponding joint and segment angles. Leg length was obtained as sum of average thigh and shank lengths which were taken by the digitized data across the whole stride cycle in reference to a previous study (Toyoshima and Sakurai, 2016). In reference to variables used in previous studies (Kunz and Kaufmann, 1981; Alexander, 1989; Ae et al., 1992; Hunter et al., 2004; Bushnell and Hunter, 2007; Ito et al., 2008; Yada et al., 2011; Toyoshima and Sakurai, 2016; Haugen et al., 2018), the kinematic variables listed in Table 1 were extracted.

**Table 1:** Variables used in this study and descriptive statistics for each one based on the studied cohort.

**Variables [units]**		**Mean**	**SD**	**Min**.	**Max**.
Age [years]		20.7	1.9	18.0	27.0
Stature [m]		1.75	0.05	1.62	1.85
Body mass [kg]		66.6	5.0	48.4	79.0
100-m personal best time [s]		11.08	0.42	10.30	12.14
Leg length [m]		0.812	0.032	0.732	0.885
Spatiotemporal variables	Running speed [m/s]	9.90	0.46	8.83	10.97
	Step length [m]	2.15	0.11	1.88	2.37
	Step frequency [Hz]	4.60	0.22	4.17	5.17
	Stride time [s]	0.435	0.020	0.387	0.480
	Left support time [s]	0.103	0.007	0.087	0.120
	Left flight time [s]	0.112	0.009	0.093	0.133
	Right support time [s]	0.105	0.007	0.093	0.120
	Right flight time [s]	0.115	0.010	0.093	0.133
	Right swing time [s]	0.330	0.017	0.287	0.373
	Swing/support ratio	3.16	0.24	2.71	3.71
	Flight/support ratio	1.10	0.11	0.88	1.41
Swing leg kinematics	Thigh angle at contralateral foot strike [deg]	4.1	8.6	−17.7	22.9
	Minimum knee joint angle [deg]	31.6	5.6	22.1	47.3
	Maximal thigh lift angle [deg]	70.3	4.6	62.0	83.6
	Maximal knee flexion angular velocity [deg/s]	−1,185	92	−1,397	−874
	Maximal thigh lift angular velocity [deg/s]	792	47	641	887
	Maximal leg backward swing angular velocity [deg/s]	−466	50	−569	−349
Support leg kinematics	Relative foot strike distance (anteroposterior distance between hip and the fifth metatarsal head at foot strike/leg length ×100) [%]	49.8	3.6	39.7	56.9
	Relative toe-off distance (anteroposterior distance between hip and the fifth metatarsal head at toe-off/leg length ×100) [%]	72.4	3.9	62.6	83.0
	Hip angle at foot strike [deg]	131.9	3.7	123.7	140.0
	Knee angle at foot strike [deg]	152.3	5.6	140.3	166.5
	Ankle angle at foot strike [deg]	123.2	4.3	112.8	133.6
	Hip angle at toe-off [deg]	196.4	5.3	182.2	209.6
	Knee angle at toe-off [deg]	155.4	4.8	141.5	168.2
	Ankle angle at toe-off [deg]	147.3	4.5	136.3	156.7
	Knee flexion angular displacement [deg]	−13.6	3.6	−3.8	−20.9
	Ankle dorsiflexion angular displacement [deg]	−19.1	3.9	−7.6	−28.7
	Hip extension angular displacement [deg]	64.5	5.1	48.7	74.9
	Knee extension angular displacement [deg]	16.9	5.7	2.6	32.6
	Ankle plantarflexion angular displacement [deg]	43.2	3.8	36.2	55.7
	Maximal hip extension velocity [deg/s]	850	73	615	992
	Maximal knee extension velocity [deg/s]	443	118	98	726
	Maximal ankle plantarflexion velocity [deg/s]	1,009	92	798	1,236
	Thigh angle at foot strike [deg]	33.0	3.6	24.0	40.2
	Shank angle at foot strike [deg]	5.3	3.2	−3.0	14.5
	Foot angle at foot strike [deg]	62.1	3.6	53.7	69.4
	Thigh angle at toe-off [deg]	−28.7	3.6	−37.6	−15.7
	Shank angle at toe-off [deg]	−53.3	2.9	−61.0	−46.4
	Foot angle at toe-off [deg]	−20.6	4.7	−32.0	−10.1
	Thigh angular displacement [deg]	61.8	5.1	45.3	72.4
	Shank angular displacement [deg]	58.6	3.6	50.4	67.7
	Foot angular displacement [deg]	82.8	4.4	72.2	93.7
	Maximal leg backward swing angular velocity [deg/s]	−664	43	−751	−572

### Statistical Analyses

Simple linear regression analysis was used to test the relationship between stature (independent variable) and leg length (dependent variable), between swing/support ratio (independent variable) and flight/support ratio (dependent variable), and between running speed (independent variable) and leg length (dependent variable). Multiple linear regression analysis was used to examine the relationship of running speed and leg length (independent variables) with step frequency (dependent variable), of running speed, leg length, and step frequency (independent variables) with swing/support ratio (dependent variable), and of running speed, leg length, step frequency, and swing/support ratio (independent variables) with each of the kinematic variables (dependent variable). The significance level was *p* <0.05. Threshold values for the interpretation of the adjusted *R*^2^ as an effect size were set at 0.02 (small), 0.13 (medium), 0.26 (large) in accordance with Cohen (1988). All statistical values were calculated using SPSS statistical software (IBM, Tokyo, Japan). To evaluate the magnitudes of changes in kinematic variables with changes in each independent variable, the running speed, leg length, step frequency and swing/support ratio were manipulated using obtained regression equation in reference to a previous study (Hunter et al., 2004). The inputs were the mean and 2 standard deviation (SD) or 2 standard error of estimate (SEE) values for running speed and leg length or for step frequency and swing/support ratio. The 2 SD or 2 SEE was selected because 2 SD indicates that 95.45% of values lie within a band around the mean in a normal distribution. That is, using the range of 2 SD or 2 SEE covers changes in kinematics associated with realistic changes in running speed and leg length or step frequency and swing/support ratio. For the manipulation, variables with a medium or large effect size (based on adjusted *R*^2^ > 0.13) were selected. The magnitudes of changes in kinematic variables with the manipulation were expressed as a ratio (percentage) in relation to mean value of each kinematic variable.

## Results

There were significant correlations between stature and leg length (*r* = 0.843, *p* < 0.001) and between swing/support ratio and flight/support ratio (*r* = 0.916, *p* < 0.001) (Table 2), while running speed was not correlated with leg length (*r* = 0.186, *p* = 0.100). Running speed and leg length combined in a significant regression model to predict step frequency (adjusted *R*^2^ = 0.382, large effect). Running speed, leg length and step frequency combined in a significant regression model to predict swing/support ratio (adjusted *R*^2^ = 0.183, medium effect).

**Table 2:** Multiple regression equations to calculate leg length, flight/support ratio, step frequency, and swing/support ratio.

**Dependent variables [units]**	**Multiple regression equations**	***p***	**SEE**	***R***	***R*^**2**^**
Leg length [m]	Y = 0.519·Stat – 0.095	<0.001	0.017	0.843	0.707
Flight/support ratio	Y = 0.431·SSR – 0.261	<0.001	0.045	0.916	0.836
Step frequency [Hz]	Y = 0.236·RS – 3.320·LL + 4.965	<0.001	0.170	0.631	0.382
Swing/support ratio	Y = 0.255·RS – 2.624·LL – 0.547·SF + 5.288	<0.001	0.212	0.463	0.183

*Stat, stature [m]; SSR, swing/support ratio; RS, running speed [m/s]; LL, leg length [m]; SF, step frequency [Hz]; SEE, standard error of estimate; R, multiple correlation coefficient; R^2^, determination coefficient adjusted for the degrees of freedom*.

For swing leg kinematics, running speed, leg length, step frequency, and swing/support ratio combined in a significant regression model to predict thigh angle at the contralateral foot strike, maximal thigh lift angle, maximal knee flexion angular velocity, maximal thigh lift angular velocity, and maximal leg backward swing velocity (adjusted *R*^2^ = 0.122–0.378, small to large effect) (Table 3). For support leg kinematics, running speed, leg length, step frequency and swing/support ratio combined in a significant regression model to predict the relative foot strike distance, relative toe-off distance, hip, knee and ankle angles at the foot strike and toe-off, hip extension angular displacement, knee flexion and extension angular displacements, maximal hip, knee and ankle extension (plantar-flexion) angular velocities, thigh and shank angles at the ipsilateral foot strike and toe-off, foot angle at the ipsilateral toe-off, thigh, shank and foot angular displacements from foot strike to toe-off, and maximal leg backward swing angular velocity (adjusted *R*^2^ = 0.074–0.757, small to large effect). For the minimum knee joint angle during the swing phase and ankle dorsi- and plantar-flexion angular displacements and foot angle at foot strike during the support phase, a significant regression was not obtained.

**Table 3:** Multiple regression equations to calculate leg kinematic variables.

	**Dependent variables [units]**	**Multiple regression equations**	***p***	**SEE**	***R***	***R*^**2**^**
Swing leg kinematics	Thigh angle at contralateral foot strike [deg]	Y = 3.59·RS + 73.38·LL + 11.65·SF + 8.70·SSR – 172.25	<0.001	7.73	0.492	0.201
	Maximal thigh lift angle [deg]	Y = 4.57·RS + 2.07·LL – 6.20·SF – 2.45·SSR + 59.67	0.008	4.33	0.408	0.122
	Maximal knee flexion angular velocity [deg/s]	Y = −45.66·RS + 1165.12·LL – 96.13·SF + 40.61·SSR – 1364.41	<0.001	76.38	0.588	0.310
	Maximal thigh lift angular velocity [deg/s]	Y = 53.33·RS – 780.62·LL – 79.02·SF – 51.98·SSR + 1425.58	<0.001	41.85	0.485	0.194
	Maximal leg backward swing angular velocity [deg/s]	Y = −49.87·RS + 443.88·LL + 19.51·SF – 75.11·SSR – 184.86	<0.001	39.72	0.641	0.378
Support leg kinematics	Relative foot strike distance [%]	Y = 4.24·RS – 57.73·LL – 11.97·SF – 11.60·SSR + 146.43	<0.001	2.51	0.729	0.507
	Relative toe-off distance [%]	Y = 6.85·RS – 68.05·LL – 11.89·SF – 11.79·SSR + 151.84	<0.001	2.76	0.732	0.510
	Hip angle at foot strike [deg]	Y = −2.21·RS + 12.15·LL + 4.29·SF + 7.86·SSR + 99.31	0.002	3.39	0.450	0.159
	Knee angle at foot strike [deg]	Y = – 0.51·RS + 30.25·LL – 1.87·SF + 8.89·SSR + 113.24	0.005	5.21	0.422	0.134
	Ankle angle at foot strike [deg]	Y = 1.79·RS + 10.68·LL – 7.39·SF + 0.52·SSR + 129.15	0.014	4.11	0.391	0.107
	Hip angle at toe-off [deg]	Y = 5.50·RS – 75.78·LL – 9.98·SF – 7.76·SSR + 274.00	0.003	4.85	0.442	0.152
	Knee angle at toe-off [deg]	Y = 1.69·RS – 50.03·LL – 9.99·SF – 1.30·SSR + 229.36	0.037	4.62	0.357	0.080
	Ankle angle at toe-off [deg]	Y = 2.21·RS – 2.41·LL – 8.46·SF – 1.22·SSR + 170.17	0.046	4.36	0.348	0.074
	Knee flexion angular displacement [deg]	Y = −1.33·RS + 11.32·LL + 9.66·SF + 4.38·SSR – 68.00	<0.001	3.25	0.491	0.200
	Hip extension angular displacement [deg]	Y = 7.71·RS – 87.93·LL – 14.27·SF – 15.62·SSR + 174.69	<0.001	3.72	0.707	0.473
	Knee extension angular displacement [deg]	Y = 3.41·RS – 89.19·LL – 17.21·SF – 14.61·SSR + 181.06	<0.001	4.41	0.652	0.394
	Maximal hip extension velocity [deg/s]	Y = 100.74·RS – 1214.75·LL – 141.65·SF – 142.50·SSR + 1941.08	<0.001	61.95	0.568	0.286
	Maximal knee extension velocity [deg/s]	Y = 82.39·RS – 1970.14·LL – 340.27·SF – 296.98·SSR + 3732.32	<0.001	94.19	0.627	0.360
	Maximal ankle plantarflexion velocity [deg/s]	Y = 50.30·RS – 703.02·LL – 41.65·SF + 62.17·SSR + 1076.94	0.042	87.94	0.352	0.077
	Thigh angle at foot strike [deg]	Y = 2.18·RS – 37.32·LL – 4.89·SF – 10.76·SSR + 98.26	<0.001	2.82	0.640	0.378
	Shank angle at foot strike [deg]	Y = 1.67·RS – 7.07·LL – 6.75·SF – 1.87·SSR + 31.50	0.035	3.10	0.359	0.081
	Thigh angle at toe-off [deg]	Y = −4.02·RS + 64.31·LL + 10.12·SF + 7.17·SSR – 110.38	<0.001	3.04	0.557	0.273
	Shank angle at toe-off [deg]	Y = −2.33·RS + 14.28·LL + 0.13·SF + 5.87·SSR – 61.02	<0.001	2.57	0.517	0.228
	Foot angle at toe-off [deg]	Y = −4.54·RS + 16.69·LL + 8.60·SF + 7.08·SSR – 51.19	0.006	4.38	0.416	0.129
	Thigh angular displacement [deg]	Y = 6.20·RS – 101.63·LL – 15.01·SF – 17.92·SSR + 208.64	<0.001	3.25	0.785	0.595
	Shank angular displacement [deg]	Y = 4.00·RS – 21.35·LL – 6.89·SF – 7.73·SSR + 92.52	<0.001	3.18	0.507	0.217
	Foot angular displacement [deg]	Y = 4.42·RS – 34.44·LL – 7.96·SF – 9.46·SSR + 133.53	0.001	4.00	0.478	0.187
	Maximal leg backward swing angular velocity [deg/s]	Y = −61.31·RS + 853.19·LL – 16.52·SF – 4.39·SSR – 659.85	<0.001	21.23	0.877	0.757

*RS, running speed [m/s]; LL, leg length [m]; SF, step frequency [Hz]; SSR, swing/support ratio; SEE, standard error of estimate; R, multiple correlation coefficient; R^2^, determination coefficient adjusted for the degrees of freedom*.

Table 4 shows four examples of 21 selected leg kinematic variables (i.e., those with a medium or large adjusted *R*^2^) when each of the predictors changes. Comparing the changes in the values of the predicted kinematic variables among the four conditions with the same magnitude of changes in predictors (i.e., ±2SD for condition A and B, ±2SEE for condition C and D), the greatest changes were found in condition A for thigh angle at contralateral foot strike and maximal leg backward swing velocities during the swing and support phases (3 variables), in condition B for maximal knee flexion angular velocity and maximal thigh lift angular velocity (2 variables), in condition C for knee flexion angular displacement (1 variables), and in condition D for the rest of variables (15 variables).

**Table 4:** Examples of changes in predicted leg kinematic variables for four conditions.

		**Condition A**			**Condition B**			**Condition C**			**Condition D**			**Magnitude of change [%]**			
		**(a)**	**(b)**	**(c)**	**(d)**	**(e)**	**(f)**	**(g)**	**(h)**	**(i)**	**(j)**	**(k)**	**(l)**	**(c)–(a)**	**(f)–(d)**	**(i)–(g)**	**(l)–(j)**
Running speed [m/s]		**8.99**	**9.90**	**10.82**	9.90			9.90			9.90			**18.5**	0.0	0.0	0.0
Leg length [m]		0.812			**0.749**	**0.812**	**0.875**	0.812			0.812			0.0	**15.6**	0.0	0.0
Step frequency [Hz]		4.39	4.60	4.82	4.81	4.60	4.39	**4.27**	**4.60**	**4.94**	4.60			9.4	−9.1	**14.7**	0.0
Swing/support ratio		3.05	3.16	3.28	3.21	3.16	3.11	3.35	3.16	2.98	**2.74**	**3.16**	**3.59**	7.3	−3.2	−11.8	**26.9**
Swing leg kinematics	Thigh angle at contralateral foot strike [deg]	−2.8	4.1	10.9	2.3	4.1	5.8	1.7	4.1	6.4	0.4	4.1	7.8	335.4	86.2	114.9	181.7
	Maximal knee flexion angular velocity [deg/s]	−1,127	−1,185	−1,243	−1,277	−1,185	−1,093	−1,145	−1,185	−1,225	−1,202	−1,185	−1,167	9.8	−15.5	6.8	−2.9
	Maximal thigh lift angular velocity [deg/s]	766	792	817	822	792	761	809	792	774	814	792	770	6.5	−7.6	−4.3	−5.6
	Maximal leg backward swing velocity [deg/s]	−416	−466	−516	−494	−466	−438	−486	−466	−445	−434	−466	−498	21.5	−12.0	−8.8	13.7
Support leg kinematics	Relative foot strike distance [%]	49.8	49.8	49.8	50.3	49.8	49.2	51.7	49.8	47.9	54.7	49.8	44.9	−0.1	−2.2	−7.7	−19.8
	Relative toe-off distance [%]	70.0	72.4	74.7	73.6	72.4	71.2	74.2	72.4	70.6	77.4	72.4	67.4	6.5	−3.3	−5.1	−13.8
	Hip angle at foot strike [deg]	132.1	131.9	131.7	132.4	131.9	131.4	131.9	131.9	131.9	128.6	131.9	135.2	−0.3	−0.8	0.0	5.1
	Knee angle at foot strike [deg]	152.1	152.3	152.4	150.4	152.3	154.1	154.6	152.3	150.0	148.5	152.3	156.1	0.2	2.4	−3.0	5.0
	Hip angle at toe-off [deg]	194.4	196.4	198.4	198.7	196.4	194.1	198.4	196.4	194.5	199.7	196.4	193.1	2.0	−2.3	−2.0	−3.4
	Knee flexion angular displacement [deg]	−15.0	−13.6	−12.2	−12.1	−13.6	−15.2	−16.1	−13.6	−11.2	−15.5	−13.6	−11.8	−20.2	22.6	−36.2	−27.3
	Hip extension angular displacement [deg]	62.4	64.5	66.7	66.3	64.5	62.8	66.5	64.5	62.6	71.2	64.5	57.9	6.8	−5.5	−6.0	−20.6
	Knee extension angular displacement [deg]	19.2	16.9	14.6	18.2	16.9	15.6	20.1	16.9	13.8	23.1	16.9	10.7	−27.0	−15.2	−36.9	−73.3
	Maximal hip extension velocity [deg/s]	804	850	895	889	850	810	871	850	828	910	850	789	10.7	−9.4	−5.1	−14.3
	Maximal knee extension velocity [deg/s]	475	443	411	481	443	405	503	443	383	569	443	317	−14.6	−17.2	−27.2	−57.0
	Thigh angle at foot strike [deg]	33.3	33.0	32.7	33.8	33.0	32.3	32.7	33.0	33.4	37.6	33.0	28.5	−1.8	−4.8	2.1	−27.7
	Thigh angle at toe-off [deg]	−28.0	−28.7	−29.4	−30.3	−28.7	−27.1	−30.8	−28.7	−26.6	−31.8	−28.7	−25.7	4.7	−11.0	−14.6	−21.2
	Shank angle at toe-off [deg]	−51.9	−53.3	−54.8	−53.9	−53.3	−52.7	−52.3	−53.3	−54.4	−55.8	−53.3	−50.8	5.4	−2.2	3.9	−9.3
	Thigh angular displacement [deg]	61.4	61.8	62.1	64.1	61.8	59.4	63.5	61.8	60.0	69.4	61.8	54.1	1.2	−7.7	−5.7	−24.7
	Shank angular displacement [deg]	57.4	58.6	59.9	58.2	58.6	59.1	59.5	58.6	57.7	61.9	58.6	55.4	4.4	1.7	−3.1	−11.2
	Foot angular displacement [deg]	81.5	82.8	84.0	82.8	82.8	82.7	83.7	82.8	81.8	86.8	82.8	78.7	3.0	−0.1	−2.3	−9.7
	Maximal leg backward swing angular velocity [deg/s]	−604	−664	−724	−722	−664	−606	−659	−664	−669	−662	−664	−666	18.2	−17.4	1.4	0.6

*Condition A: Predicted kinematic variables when the sprinter's leg length was 0.812 m (mean value in this study) and running speeds were 8.99, 9.90, and 10.82 m/s (mean ± 2SD values in this study). The values of step frequency were calculated using the regression equation presented in Table 2 with running speeds and leg length, while the values of the swing/support ratio were computed using the regression equation presented in Table 2 with running speeds, leg length, and predicted step frequencies*.

*Condition B: Predicted kinematic variables when running speed was 9.90 m/s (mean value in this study) and the sprinter's leg lengths were 0.749, 0.812, and 0.875 m (mean ± 2SD values in this study). The values of step frequency were calculated using the regression equation presented in Table 2 with running speed and leg lengths, while the values of the swing/support ratio were computed using the regression equation presented in Table 2 with running speed, leg lengths, and predicted step frequencies*.

*Condition C: Predicted kinematic variables when running speed was 9.90 m/s, the sprinter's leg length was 0.812, and step frequencies were 4.27, 4.60, and 4.94 Hz (mean ± 2SEE values in this study). The values of the swing/support ratio were computed using the regression equation presented in Table 2 with running speed, leg length, and step frequencies*.

*Condition D: Predicted kinematic variables when running speed was 9.90 m/s, the sprinter's leg length was 0.812, step frequency was 4.60 Hz, and swing/support ratio were 2.74, 3.16, and 3.59 (mean ± 2SEE values in this study)*.

*Bold numbers indicate manipulated predictor variables*.

## Discussion

This study aimed to provide multiple regression equations taking into account differences in running speed, leg length and step characteristics to predict kinematics of maximal speed sprinting for understanding kinematics of faster maximal speed sprinting with the difference in leg length and spatiotemporal variables. Employing a large number (*n* = 79) of sprinters across a broad range of performance levels (10.30–12.14 s), multiple regression equations which took into account difference in running speed, leg length and spatiotemporal variables to predict kinematics of maximal speed sprinting were successfully obtained, and leg kinematics of greater maximal running speed based on leg length and step characteristics were elucidated using the multiple regression equations. Although there were previous studies that examined the relationship between running speed and each of kinematic variables (Kunz and Kaufmann, 1981; Alexander, 1989; Ae et al., 1992; Bushnell and Hunter, 2007; Ito et al., 2008; Yada et al., 2011; Toyoshima and Sakurai, 2016; Haugen et al., 2018), this study is the first to demonstrate kinematic features for faster sprinting performance, taking into account the characteristics of individuals in terms of leg length and spatiotemporal variables. Moreover, as the adjusted *R*^2^ for all predicted kinematic variables were greater than *R*^2^ for each of simple linear regression analyses (Supplementary Table 1), it is evident that not only running speed, but also leg length and spatiotemporal variables (step frequency and swing/support ratio), relate to leg kinematics.

Taking into account the significant correlations for stature and leg length, for swing/support ratio and flight/support ratio, and not for running speed and leg length, the regressions among the running speed, leg length, step frequency, and swing/support ratio demonstrate that faster running speed is associated with higher step frequency and greater swing (flight)/support ratio regardless of leg length (stature). The significant relationship for running speed and step frequency and not for running speed and leg length are supported by previous studies which employed a large number of participants (Ito et al., 2008; Nagahara et al., 2018b). Moreover, in line with a previous study (Nagahara et al., 2018b), the results indicate that the longer the leg length, the lower the step frequency and swing/support ratio, while the higher the step frequency, the lower the swing/support ratio. As moment of inertia theoretically increases with the square of the length for a given mass, a long leg length will make it difficult to rotate fast, resulting in a decrease in step frequency. In addition, a long leg length at a given running speed and step frequency will theoretically lead to long support time with long support distance. Because step frequency is an inverse of step time which consists of support and flight times, and support time at a given speed and leg length is difficult to change due to geometric constraints, higher step frequency through shorter step and flight times will be accompanied with lower swing/support ratio. Accordingly, it can be said that the aforementioned findings are theoretically reasonable.

Relative foot strike distance, hip, knee, and thigh angles at foot strike, hip angle at toe-off, and thigh angular displacement showed small percentage changes (<2%) in association with changes in running speed of ±2SD (Table 4). Thus, the influence of changes in running speed on these variables can be considered as negligible. For faster maximal speed sprinting with the same leg length, greater thigh angle at the contralateral foot strike, maximal knee flexion and thigh lift angular velocities, and maximal leg backward swing velocity can be considered as important kinematic features during the swing phase. While some important variables cannot be compared with previous studies, the importance of thigh angle at the contralateral foot strike and maximal leg backward velocity has been confirmed in previous studies (Ae et al., 1992; Bushnell and Hunter, 2007; Yada et al., 2011). Greater thigh lift angle at the contralateral foot strike and faster thigh lift angular velocity indicate faster recovery of the swing leg, and this motion can assist in the rapid production of vertical force, through upward acceleration of the swing leg that is essential for achieving high maximal speed sprinting (Weyand et al., 2000). Foot velocity in relation to the body center of mass during the support phase is equal to running speed, and as the whole leg angular velocity is one of the mechanical determinants of foot velocity, these results appear logical.

During the support phase, greater relative toe-off distance, smaller knee flexion and extension angular displacements, greater hip extension angular displacement, greater maximal hip extension and smaller maximal knee extension velocities, greater thigh and shank forward lean angles at toe-off, greater shank and foot angular displacements, and greater maximal leg backward swing velocity were defined as essential kinematic features for faster maximal speed sprinting with the same leg length based on magnitudes of the changes (>2%). The following kinematic features are in line with previous studies: smaller knee flexion angular displacement (Yada et al., 2011), smaller knee extension angular displacement (Yada et al., 2011), greater hip extension velocity (Ae et al., 1992; Ito et al., 2008), smaller knee extension velocity (Ae et al., 1992; Ito et al., 2008), greater shank angular displacement (Alexander, 1989), and greater maximal leg backward swing velocity (Ae et al., 1992) during the support phase. For the kinematic variables related to the first half of the support phase, only the knee flexion angular displacement showed a large change (>2%) when running speed was increased. Just after foot strike, it is important to produce vertical force rapidly for high maximal speed sprinting (Clark and Weyand, 2014), and knee flexion during the first half of the support phase would suppress the production of the vertical force. Thus, the importance of producing vertical force rapidly during the initial support phase possibly explains the relationship between running speed and the knee flexion range. Greater relative toe-off distance, greater forward lean thigh and shank at toe-off, and greater hip, shank, and foot angular displacements during the support phase are all indicative of a more forward leaning leg position during the second half of the support phase. Although it is difficult to provide a clear rationale for the importance of these kinematic features for greater running speed, one possible reason is that a forward leaning leg posture is likely to facilitate the production of propulsive force (Kugler and Janshen, 2010), while this was determined during early acceleration and the importance of producing propulsive force disappears by the maximal speed phase (Nagahara et al., 2018a). As mentioned above, foot velocity in relation to the body center of mass is equal to running speed during the support phase, and the leg angular velocity is mechanically one of the determinants of this foot velocity, with a greater hip extension velocity likely increasing this leg angular velocity. As knee extension would reduce the leg backward swing velocity during the support phase (Ito et al., 2008), increasing hip extension and suppressing knee extension velocities are again logical techniques for faster maximal speed sprinting through the role in facilitating higher leg backward swing velocity during the support phase.

The inter-individual differences in leg length (stature) have influence on leg kinematics for running at a specific speed (Table 4). When compared to the magnitudes of changes in kinematic variables in association with changes in running speed over ±2SD, corresponding magnitudes in association with changes in leg length over ±2SD were greater for 11 out of 21 variables. The fact that the difference in leg length has a comparable or greater influence on running kinematics in comparison with the differences in running speed demonstrates the importance of considering leg length for examining the kinematics of faster maximal speed sprinting. The knowledge gained in the current study is useful for considering the effects of differences in sprinters' leg lengths. Although there is no previous study against which a direct comparison can be made, Nagahara et al. (2018b) reported that greater stature was associated with lower step frequency and longer support time during the maximal speed sprinting, thus partially supporting the current findings. Based on the obtained regression equations with major kinematic changes, longer-legged sprinters will accomplish the same running speed with a lower step frequency, a greater thigh angle at contralateral foot strike, smaller maximal knee flexion velocity during the swing phase, smaller leg backward swing velocities during the swing and support phases, greater flexion and smaller extension ranges of knee joint during the support phase, and smaller thigh forward lean at toe-off.

At a given running speed and leg length, based on the obtained regression equations with major kinematic changes, higher step frequencies will be achieved with a lower swing/support ratio, a greater thigh angle at contralateral foot strike, smaller knee flexion and extension ranges during the support phase, smaller maximal knee extension velocity, and smaller thigh forward lean angle at toe-off (Table 4). Trying to recover the swing leg earlier and to suppress changes in knee joint angle during the support phase therefore may result in increases in step frequency. At a given running speed, leg length, and step frequency, based on the obtained regression equations with major kinematic changes, a greater swing/support ratio will be accomplished with a greater thigh angle at contralateral foot strike, smaller hip extension, knee flexion and extension ranges during the support phase, smaller maximal knee extension velocity during the support phase, smaller thigh angles at foot strike and toe-off (both close to the upright position), and smaller thigh angular displacement during the support phase (Table 4). Trying to recover the swing leg earlier and to suppress changes in knee joint angle with a small range of thigh motion during the support phase will therefore raise the swing/support ratio.

Using running speed, leg length, and spatiotemporal variables which can be collected using smartphone in addition to the regression equations obtained in this study, a model of leg kinematics during the maximal speed sprinting can be provided. Although angular velocities are difficult to obtain for practitioners, joint angles can be measured using freely-available software (e.g., Kinovea) to analyse images from an appropriately positioned video camera. This will make it possible to compare the model leg kinematic features for specific running speed with the current kinematic features of a sprinter. Consequently, the regression equations in this study will be useful for sprinters and coaches when trying to improve leg kinematics for achieving higher maximal running speed.

Regarding the limitations of the current study, the participants employed in this study ranged from 10.30 to 12.14 s. Thus, the obtained regression equations are appropriate for the range of sprinters' performance level used in this study, and it is possible that the results might differ when sprinters with smaller range of performance levels are employed. Because we did not use multiple cameras to obtain three dimensional coordinates of body segments, influences of running speed, leg length, and spatiotemporal variables on leg kinematics in the coronal and transverse planes during maximal speed sprinting are still unknown. As the locations of the body landmarks were manually digitized and the foot strike and toe-off instants were visually detected, an investigation using a motion capture system which consists of infra-red cameras and force platforms will possibly derive different results compared to the current results. There was a variation of adjusted *R*^2^ values among multiple regression equations, and this indicates that there would be other variables which have influences on the kinematics of maximal speed sprinting. For some variables, even if there was a medium effect size (adjusted *R*^2^ > 0.13), the adjusted *R*^2^ value indicates that the multiple regression equation can partially (>13%) explain the changes in a kinematic variable. Because this was a cross-sectional study as the regression equations were extracted using data from 79 sprinters, it is possible that intra-individual changes in kinematic variables associated with changes in running speed, step frequency, and swing/support ratio are not consistent to the predicted changes using the multiple regression equations. Although we instructed participants to achieve their maximal speed during the section from the 40- to 50-m mark, it is possible that the exact maximal sprint speed was not appeared within the section from 40 to 50-m mark for some participants because we did not measure consecutive running speed from the start of the trial. However, the running speed and modality only slightly changes around the maximal speed in sprinting (Nagahara et al., 2014a; Slawinski et al., 2017), and thus it can be considered that the influence of difference in locations of maximal speeds is negligible as previous studies adopted the same locations for investigating kinematics and kinetics of maximal speed sprinting (Alexander, 1989; Bushnell and Hunter, 2007; Bezodis et al., 2008; Yada et al., 2011). Although this study was performed with male sprinters, Ciacci et al. (2017) clarified that kinematics of sprinting was only partially affected by the sex of sprinters, and the differences in kinematics were mainly produced by the difference in performance level. Therefore, there is the possibility that the findings in this study may translate to female sprinters as long as they are within the studied performance levels.

In conclusion, employing a large number (*n* = 79) of sprinters over a relatively wide range of performance levels (10.30–12.14 s), multiple regression equations taking into account differences in running speed, leg length, and step characteristics to predict kinematics of maximal speed sprinting were successfully obtained, and leg kinematic features of faster maximal speed sprinting at different leg length and step characteristics were elucidated using the regression equations. The regression equations obtained in this study will be useful for sprinters and coaches when trying to improve their maximal speed sprinting motion based on the specific target changes in running speed and spatiotemporal variables for individuals with different leg lengths.

## Data Availability Statement

The datasets generated for this study will be made available by the authors, after explicit and justified request, to any qualified researcher.

## Ethics Statement

This studies involving human participants were reviewed and approved by Research ethics committee of the Faculty of Health and Sports Sciences, University of Tsukuba (#22-409). The patients/participants provided their written informed consent to participate in this study.

## Author Contributions

KM, RN, KY, and TN contributed to conceiving, designing, performing the experiment, analyzing the data, drafting, and revising the article. KM performed most of the data analysis. RN performed most of drafting the article.

### Conflict of Interest

The authors declare that the research was conducted in the absence of any commercial or financial relationships that could be construed as a potential conflict of interest.
