# Supplementary material for: Kinematics of Maximal Speed Sprinting With Different Running Speed, Leg Length, and Step Characteristics
Source: Front Sports Act Living. 2019 Sep 26;1:37. doi: 10.3389/fspor.2019.00037 (PMC7739839; doi:10.3389/fspor.2019.00037)
Supplement: Supplementary file 1 [file Table_1.docx]

Suppl. 1 Results of multiple regression analysis for each predicted variable and predictors.

| Independent variables | Dependent variables | SEB | *β* | P(*β*) | r | r^2^ | r_xy･z_ |
| --- | --- | --- | --- | --- | --- | --- | --- |
| Thigh angle at the contralateral foot strike | Running speed | 2.54 | .191 | .161 | .413 | .171 | .163 |
|  | Leg length | 34.84 | .269 | .039 | .175 | .031 | .238 |
|  | Step frequency | 5.71 | .291 | .045 | .226 | .051 | .231 |
|  | Swing/support ratio | 4.20 | .236 | .042 | .216 | .047 | .234 |
|  | (constant) | 43.53 |  |  |  |  |  |
| Maximal thigh lift angle | Running speed | 1.42 | .454 | .002 | .310 | .096 | .350 |
|  | Leg length | 19.50 | .014 | .916 | .221 | .049 | .012 |
|  | Step frequency | 3.20 | −.290 | .056 | −.089 | .008 | −.220 |
|  | Swing/support ratio | 2.35 | −.125 | .301 | .023 | .001 | −.120 |
|  | (constant) | 24.37 |  |  |  |  |  |
| Maximal knee flexion angular velocity | Running speed | 25.07 | −.228 | .073 | −.222 | .049 | −.207 |
|  | Leg length | 344.35 | .401 | .001 | .441 | .194 | .366 |
|  | Step frequency | 56.43 | −.226 | .093 | −.494 | .244 | −.194 |
|  | Swing/support ratio | 41.51 | .104 | .331 | .063 | .004 | .113 |
|  | (constant) | 430.29 |  |  |  |  |  |
| Maximal thigh lift angular velocity | Running speed | 13.74 | .525 | <.001 | .217 | .047 | .411 |
|  | Leg length | 188.70 | −.530 | <.001 | −.272 | .074 | −.433 |
|  | Step frequency | 30.93 | −.366 | .013 | .100 | .010 | −.285 |
|  | Swing/support ratio | 22.75 | −.262 | .025 | −.052 | .003 | −.257 |
|  | (constant) | 235.79 |  |  |  |  |  |
| Maximal leg backward swing velocity | Running speed | 13.04 | −.454 | <.001 | −.447 | .199 | −.406 |
|  | Leg length | 179.08 | .279 | .015 | .184 | .034 | .277 |
|  | Step frequency | 29.35 | .084 | .508 | −.157 | .025 | .077 |
|  | Swing/support ratio | 21.59 | −.350 | <.001 | −.483 | .234 | −.375 |
|  | (constant) | 223.78 |  |  |  |  |  |
| Relative foot strike distance | Running speed | 0.82 | .544 | <.001 | −.019 | <.001 | .513 |
|  | Leg length | 11.32 | −.511 | <.001 | −.078 | .006 | −.510 |
|  | Step frequency | 1.86 | −.722 | <.001 | −.176 | .031 | −.600 |
|  | Swing/support ratio | 1.37 | −.762 | <.001 | −.493 | .243 | −.703 |
|  | (constant) | 14.15 |  |  |  |  |  |
| Relative toe-off distance | Running speed | 0.91 | .796 | <.001 | .269 | .072 | .660 |
|  | Leg length | 12.46 | −.546 | <.001 | −.098 | .010 | −.536 |
|  | Step frequency | 2.04 | −.650 | <.001 | .004 | <.001 | −.560 |
|  | Swing/support ratio | 1.50 | −.702 | <.001 | −.385 | .148 | −.674 |
|  | (constant) | 15.57 |  |  |  |  |  |
| Hip angle at the foot strike | Running speed | 1.11 | −.274 | .051 | −.039 | .002 | −.225 |
|  | Leg length | 15.29 | .104 | .429 | −.077 | .006 | .092 |
|  | Step frequency | 2.51 | .250 | .091 | .017 | .000 | .195 |
|  | Swing/support ratio | 1.84 | .499 | <.001 | .391 | .153 | .444 |
|  | (constant) | 19.10 |  |  |  |  |  |
| Knee angle at the foot strike | Running speed | 1.71 | −.042 | .767 | .045 | .002 | −.035 |
|  | Leg length | 23.50 | .171 | .202 | .168 | .028 | .148 |
|  | Step frequency | 3.85 | −.072 | .630 | −.216 | .046 | −.056 |
|  | Swing/support ratio | 2.83 | .373 | .002 | .364 | .133 | .343 |
|  | (constant) | 29.36 |  |  |  |  |  |
| Ankle angle at the foot strike | Running speed | 1.35 | .189 | .188 | .059 | .004 | .153 |
|  | Leg length | 18.53 | .078 | .566 | .255 | .065 | .067 |
|  | Step frequency | 3.04 | −.366 | .017 | −.324 | .105 | −.272 |
|  | Swing/support ratio | 2.23 | .028 | .818 | .124 | .015 | .027 |
|  | (constant) | 23.15 |  |  |  |  |  |
| Hip angle at the toe-off | Running speed | 1.59 | .479 | <.001 | .148 | .022 | .373 |
|  | Leg length | 21.86 | −.456 | <.001 | −.184 | .034 | −.374 |
|  | Step frequency | 3.58 | −.409 | .007 | .022 | .001 | −.308 |
|  | Swing/support ratio | 2.63 | −.346 | .004 | −.145 | .021 | −.324 |
|  | (constant) | 27.32 |  |  |  |  |  |
| Knee angle at the toe-off | Running speed | 1.52 | .161 | .267 | −.098 | .010 | .129 |
|  | Leg length | 20.81 | −.329 | .019 | −.119 | .014 | −.269 |
|  | Step frequency | 3.41 | −.448 | .005 | −.242 | .058 | −.322 |
|  | Swing/support ratio | 2.51 | −.064 | .605 | .065 | .004 | −.060 |
|  | (constant) | 26.00 |  |  |  |  |  |
| Ankle angle at the toe-off | Running speed | 1.43 | .224 | .126 | .041 | .002 | .177 |
|  | Leg length | 19.65 | −.017 | .903 | .188 | .035 | −.014 |
|  | Step frequency | 3.22 | −.403 | .010 | −.294 | .087 | −.292 |
|  | Swing/support ratio | 2.37 | −.063 | .609 | .053 | .003 | −.060 |
|  | (constant) | 24.55 |  |  |  |  |  |
| Knee flexion angular displacement | Running speed | 1.07 | −.168 | .217 | .150 | .023 | −.143 |
|  | Leg length | 14.64 | .099 | .442 | −.176 | .031 | .090 |
|  | Step frequency | 2.40 | .574 | <.001 | .421 | .177 | .424 |
|  | Swing/support ratio | 1.76 | .283 | .015 | .148 | .022 | .277 |
|  | (constant) | 18.29 |  |  |  |  |  |
| Hip extension angular displacement | Running speed | 1.22 | .691 | <.001 | .181 | .033 | .592 |
|  | Leg length | 16.75 | −.544 | <.001 | −.133 | .018 | −.521 |
|  | Step frequency | 2.75 | −.601 | <.001 | .010 | .000 | −.517 |
|  | Swing/support ratio | 2.02 | −.717 | <.001 | −.431 | .186 | −.669 |
|  | (constant) | 20.93 |  |  |  |  |  |
| Knee extension angular displacement | Running speed | 1.45 | .276 | .021 | −.223 | .050 | .264 |
|  | Leg length | 19.87 | −.499 | <.001 | −.151 | .023 | −.463 |
|  | Step frequency | 3.26 | −.656 | <.001 | −.250 | .062 | −.524 |
|  | Swing/support ratio | 2.40 | −.606 | <.001 | −.409 | .167 | −.578 |
|  | (constant) | 24.83 |  |  |  |  |  |
| Maximal hip extension velocity | Running speed | 20.34 | .630 | <.001 | .258 | .067 | .499 |
|  | Leg length | 279.30 | −.524 | <.001 | −.214 | .046 | −.451 |
|  | Step frequency | 45.77 | −.417 | .003 | .121 | .015 | −.339 |
|  | Swing/support ratio | 33.67 | −.457 | <.001 | −.216 | .046 | −.441 |
|  | (constant) | 349.01 |  |  |  |  |  |
| Maximal knee extension velocity | Running speed | 30.92 | .321 | .009 | −.167 | .028 | .296 |
|  | Leg length | 424.65 | −.530 | <.001 | −.187 | .035 | −.475 |
|  | Step frequency | 69.59 | −.623 | <.001 | −.189 | .036 | −.494 |
|  | Swing/support ratio | 51.19 | −.593 | <.001 | −.388 | .151 | −.559 |
|  | (constant) | 530.63 |  |  |  |  |  |
| Maximal ankle plantarflexion velocity | Running speed | 28.87 | .252 | .086 | .202 | .041 | .199 |
|  | Leg length | 396.48 | −.243 | .080 | −.168 | .028 | −.202 |
|  | Step frequency | 64.98 | −.098 | .523 | .076 | .006 | −.074 |
|  | Swing/support ratio | 47.79 | .160 | .197 | .247 | .061 | .150 |
|  | (constant) | 495.42 |  |  |  |  |  |
| Thigh angle at the foot strike | Running speed | 0.93 | .279 | .021 | −.062 | .004 | .264 |
|  | Leg length | 12.72 | −.330 | .004 | −.118 | .014 | −.323 |
|  | Step frequency | 2.09 | −.295 | .022 | .062 | .004 | −.263 |
|  | Swing/support ratio | 1.53 | −.706 | <.001 | −.576 | .332 | −.632 |
|  | (constant) | 15.90 |  |  |  |  |  |
| Shank angle at the foot strike | Running speed | 1.02 | .237 | .105 | .009 | <.001 | .187 |
|  | Leg length | 13.99 | −.069 | .615 | .161 | .026 | −.059 |
|  | Step frequency | 2.29 | −.450 | .004 | −.304 | .092 | −.324 |
|  | Swing/support ratio | 1.69 | −.135 | .272 | −.006 | <.001 | −.128 |
|  | (constant) | 17.48 |  |  |  |  |  |
| Thigh angle at the toe-off | Running speed | 1.00 | −.517 | <.001 | −.053 | .003 | −.424 |
|  | Leg length | 13.72 | .571 | <.001 | .204 | .041 | .479 |
|  | Step frequency | 2.25 | .612 | <.001 | .100 | .010 | .464 |
|  | Swing/support ratio | 1.65 | .472 | <.001 | .223 | .050 | .450 |
|  | (constant) | 17.14 |  |  |  |  |  |
| Shank angle at the toe-off | Running speed | 0.84 | −.366 | .007 | −.227 | .051 | −.306 |
|  | Leg length | 11.57 | .155 | .221 | .053 | .003 | .142 |
|  | Step frequency | 1.90 | .010 | .944 | −.276 | .076 | .008 |
|  | Swing/support ratio | 1.39 | .472 | <.001 | .379 | .143 | .439 |
|  | (constant) | 14.45 |  |  |  |  |  |
| Foot angle at the toe-off | Running speed | 1.44 | −.444 | .002 | −.181 | .033 | −.345 |
|  | Leg length | 19.75 | .113 | .401 | −.148 | .022 | .098 |
|  | Step frequency | 3.24 | .395 | .010 | .112 | .013 | .295 |
|  | Swing/support ratio | 2.38 | .355 | .004 | .185 | .034 | .327 |
|  | (constant) | 24.68 |  |  |  |  |  |
| Thigh angular displacement | Running speed | 1.07 | .557 | <.001 | −.006 | <.001 | .560 |
|  | Leg length | 14.64 | −.630 | <.001 | −.225 | .051 | −.628 |
|  | Step frequency | 2.40 | −.635 | <.001 | −.026 | .001 | −.588 |
|  | Swing/support ratio | 1.76 | −.825 | <.001 | −.559 | .313 | −.763 |
|  | (constant) | 18.29 |  |  |  |  |  |
| Shank angular displacement | Running speed | 1.04 | .510 | <.001 | .192 | .037 | .407 |
|  | Leg length | 14.34 | −.188 | .141 | .102 | .010 | −.170 |
|  | Step frequency | 2.35 | −.413 | .005 | −.049 | .002 | −.322 |
|  | Swing/support ratio | 1.73 | −.505 | <.001 | −.313 | .098 | −.461 |
|  | (constant) | 17.92 |  |  |  |  |  |
| Foot angular displacement | Running speed | 1.31 | .457 | .001 | .139 | .019 | .364 |
|  | Leg length | 18.03 | −.246 | .060 | .023 | .001 | −.217 |
|  | Step frequency | 2.95 | −.387 | .009 | −.023 | .001 | −.299 |
|  | Swing/support ratio | 2.17 | −.502 | <.001 | −.322 | .103 | −.452 |
|  | (constant) | 22.53 |  |  |  |  |  |
| Maximal leg backward swing velocity | Running speed | 6.97 | −.653 | <.001 | −.576 | .332 | −.715 |
|  | Leg length | 95.72 | .628 | <.001 | .540 | .292 | .720 |
|  | Step frequency | 15.69 | −.083 | .296 | −.594 | .353 | −.122 |
|  | Swing/support ratio | 11.54 | −.024 | .705 | −.197 | .039 | −.044 |
|  | (constant) | 119.61 |  |  |  |  |  |

SEB, Standard error of the regression coefficient; *β*, Standard partial regression coefficient; r, Pearson’s correlation coefficient; r^2^, determination coefficient; r_xy･z_, Partial correlation coefficient
